# Alcohol and Viral Hepatitis: Role of Lipid Rafts

**DOI:** 10.35946/arcr.v37.2.13

**Published:** 2015

**Authors:** Angela Dolganiuc

**Affiliations:** Angela Dolganiuc, M.D., Ph.D., is a physician scientist in the Department of Medicine/Gastroenterology, Hepatology, and Nutrition at the University of Florida, Gainesville, Florida.

**Keywords:** Alcohol abuse, alcohol use and misuse, alcohol disorder, liver, liver disease, hepatitis, hepatitis B virus, hepatitis C virus, lipid rafts

## Abstract

Both alcohol abuse and infection with hepatitis viruses can lead to liver disease, including chronic hepatitis. Alcohol and hepatitis viruses have synergistic effects in the development of liver disease. Some of these involve the cellular membranes and particularly their functionally active domains, termed lipid rafts, which contain many proteins with essential roles in signaling and other processes. These lipid rafts play a central role in the lifecycles of hepatitis viruses. Alcohol’s actions at the lipid rafts may contribute to the synergistic harmful effects of alcohol and hepatitis viruses on the liver and the pathogenesis of liver disease.

Alcohol is the most used and abused psychoactive drug worldwide. Alcohol use and misuse, including alcohol use disorder, can have devastating effects and account for 5.9 percent of deaths and 5.1 percent of the global burden of disease and injury, thereby also imposing a significant social and economic burden on society (World Health Organization 2015). Moreover, treatments for alcohol abuse have shown limited effectiveness (Grant et al. 1988; National Institute on Alcohol Abuse and Alcoholism 1998). Alcohol use disorder is a systemic disease that affects all organs and systems. Evidence suggests that risk of alcohol-related organ damage occurs with excessive alcohol intake, which is defined as binge drinking or heavy drinking. According to the National Institute on Alcohol Abuse and Alcoholism, binge drinking is defined as a pattern of alcohol consumption that brings the blood alcohol concentration (BAC) level to 0.08 percent or more. This pattern of drinking usually corresponds to consumption of 5 or more drinks on a single occasion for men and 4 or more drinks on a single occasion for women, generally within about 2 hours. Heavy drinking typically is defined as consuming 15 drinks or more per week for men and 8 drinks or more per week for women (Centers for Disease Control and Prevention [CDC] 2014). The liver is particularly susceptible to alcohol-induced damage. However, although many chronic heavy drinkers develop alcoholic liver disease (ALD), no consumption levels have been identified that predictably result in ALD. Factors that influence the susceptibility to ALD include gender, co-exposure to other drugs, genetic factors that either favor the development of addiction or affect alcohol-metabolizing enzymes, immunological factors, nutritional status, and infection with viruses targeting the liver (i.e., hepatotropic viruses).

Hepatitis viruses, and particularly hepatitis B virus (HBV) and hepatitis C virus (HCV), are responsible for most cases of chronic hepatitis in the United States. In 2013, almost 20,000 new cases of HBV infection and almost 30,000 new cases of HCV infection were estimated to occur in the United States (CDC 2015*a*). Worldwide, approximately 350 to 400 million people, or about 5 percent of the population, are chronically infected with HBV and about 180 million people, or 2 percent of the population, with HCV (El-Serag 2012). In chronic alcoholics, the prevalence of HCV infection as indicated by the presence of anti-HCV antibodies is higher than in the general population (Takase et al. 1993). Co-occurring viral hepatitis and alcohol use disorder adversely affect disease course and are associated with increased mortality and death at an earlier age (Kim et al. 2001; Sagnelli et al. 2012; Tsui et al. 2006; Wiley et al. 1998). The most serious complication of ALD is liver cirrhosis, which often progresses to hepatocellular carcinoma (HCC); indeed, about 20 percent of heavy drinkers develop cirrhosis during their lifetime, and this risk is much increased in the presence of co-occurring viral hepatitis (El-Serag 2012; Ishak et al. 1991). End-stage liver disease from viral hepatitis, together with ALD, is the main reason for liver transplantation in the United States (El-Serag 2012).

The mechanisms how alcohol and viral hepatitis together accelerate liver disease have been researched extensively over the last several decades. It is becoming clear that alcohol exposure, infection with hepatitis viruses, and the host’s defense mechanisms against these offenders combine to contribute to the pathogenesis of liver disease and thus could be targets of therapeutic interventions. New antiviral drugs against HCV have been developed in recent years, and reasonably effective HBV treatments also are available (American Association for the Study of Liver Diseases 2015; Lok and McMahon 2009). Yet many patients either do not qualify for or cannot afford newer antiviral treatments. Further, exposure to alcohol, whether acute or chronic, light or heavy, may preclude eligibility for treatment of viral hepatitis. Additionally, many patients cannot achieve abstinence from alcohol or experience recurrent relapse (Becker 2008). Therefore, novel approaches are needed for the diagnosis and treatment of patients with coexisting alcohol use and viral hepatitis.

This article reviews some of the mechanisms underlying alcohol-induced liver injury and also explains the contributions of hepatitis viruses to liver disease, as well as the synergistic effects of alcohol and hepatitis virus infections on the liver. This discussion particularly focuses on the roles that the cellular membranes, and especially membrane domains called lipid rafts, play in these processes. Both alcohol and viral infections influence the functions of lipid rafts and the functional proteins they contain, which may exacerbate disease progression. The specific mechanisms underlying the effects of alcohol and hepatitis viruses on the cellular membranes and their contribution to liver disease pathogenesis, however, still remain to be fully elucidated.

## Alcohol-Induced Liver Injury

Liver injury in ALD occurs as a result of multiple synergistic mechanisms, including impaired function of the main parenchymal liver cells (i.e., hepatocytes), imbalanced local (i.e., nonparenchymal) and systemic immune responses, and altered cross-talk between parenchymal and nonparenchymal cells in the liver.

Alcohol has diverse effects on the hepatocytes that result in significant disturbances of the cells’ abilities to synthesize needed molecules and detoxify harmful products (Van Horn et al. 2001; Videla et al. 1973), pronounced deficits in antioxidant levels (Fernandez-Checa et al. 2002; Lauterburg and Velez 1988), and marked oxidative cellular stress (Tsukamoto 1993). These effects, together with additional changes in hepatocyte metabolism, lead to the accumulation of lipids in the alcohol-exposed hepatocytes (i.e., hepatic steatosis). The affected cells consume oxygen inefficiently, have reduced detoxifying ability, fail to synthesize needed compounds, and are more likely to undergo apoptosis (Nanji and Hiller-Sturmhoefel 1997). As a result of all of these changes, the cells also become more susceptible to other harmful influences, such as infections with hepatotropic viruses and dietary insufficiencies. Finally, alcohol exposure greatly enhances tumorigenesis in hepatocytes (Morgan et al. 2004).

Alcohol exposure also affects local immune responses by both hepatocytes and resident and nonresident immune cells. Hepatocytes are the first cells to encounter hepatotropic viruses, and activation of their cytokine signaling systems—including proinflammatory cytokines such as tumor necrosis factor alpha (TNF-α), interleukin (IL)-1 and IL-6, and interferons (IFNs)—is key to the initiation of immune responses. Alcohol exposure has diverse effects on these immune responses. On the one hand, alcohol suppresses intracellular expression of type I IFNs (IFN-α/β) in human hepatocytes by reducing the expression of key positive regulators of type I IFN signaling pathways and inducing the expression of key negative regulators of IFN-α/β signaling (Plumlee et al. 2005; Ye et al. 2010). On the other hand, alcohol-exposed hepatocytes increase the expression of proinflammatory TNF-α (Mandrekar 2007; Plumlee et al. 2005). In addition, alcohol exposure results in differential activation of IL-1 pathways in hepatocytes versus other nonparenchymal cells (e.g., Kupffer cells). Thus, certain active molecules (i.e., the active fragment of caspase-1 and IL-1β) are elevated only in liver immune cells but not in alcohol-exposed hepatocytes. Innate immune pathways in hepatocytes also may regulate hepatocyte steatosis and hepatocellular injury. A signaling molecule called IRF3, which is an essential component of innate immunity and is required for hepatocyte apoptosis, may play a unique role in the processes leading to hepatocyte apoptosis in ALD and tying together alcohol-induced liver inflammation, metabolic disturbances, and cell death (Petrasek et al. 2013).

Alcohol-induced cross-talk between parenchymal and nonparenchymal liver cells (e.g., Kupffer cells) is another key component of liver disease (Cohen and Nagy 2011). The activation of Kupffer cell-specific signaling pathways involving innate immune molecules called toll-like receptors (TLRs), and in particular TLR4, is emerging as a required step in the progression of liver disease from steatosis to steatohepatitis in ALD. In addition, TLR4-mediated activation of Kupffer cells seems to be important for the formation of scar tissue (i.e., fibrogenesis) in the liver after chronic alcohol treatment (Adachi et al. 1994; Inokuchi et al. 2011). Other TLRs also influence the development of ALD. Thus, alcohol exposure augments signaling via TLR8 and TLR7, thereby inducing both IL-10 and TNF-α and downgrading IFN expression in myeloid cells (Pang et al. 2011). These effects may contribute to the persistent inflammation and impaired antiviral responses in ALD. Kupffer cells seem to govern the course of ALD, especially in the early stages of the disease, because deletion of these cells protects against alcohol-induced liver injury. The mechanisms underlying these effects are not fully understood but likely are multifactorial and include cell cross-talking between innate immune cells and other liver cells, such as stellate cells (Adachi et al. 1994). Stellate cells, in turn, can develop into myofibroblasts that play a central role in alcohol-induced fibrogenesis. Alcohol exposure triggers stress signals from hepatocytes that can activate myofibroblasts, which favor excess type 1 collagen synthesis and lead to progression of fibrosis (Siegmund and Brenner 2005). Additionally, TLR4 is a key molecule involved in signaling to, from, and inside of stellate cells, suggesting that innate immune pathways also contribute to this stage of ALD (Paik et al. 2003; Seki et al. 2007).

## Hepatitis Viruses

Hepatitis viruses are a heterogeneous group of five unrelated hepatotropic viruses that cause inflammation of the liver. They include hepatitis viruses A, B, C, D, and E. Of these, HBV and HCV are clinically most relevant in Western countries.^1^

### HBV

**Figure 1:**
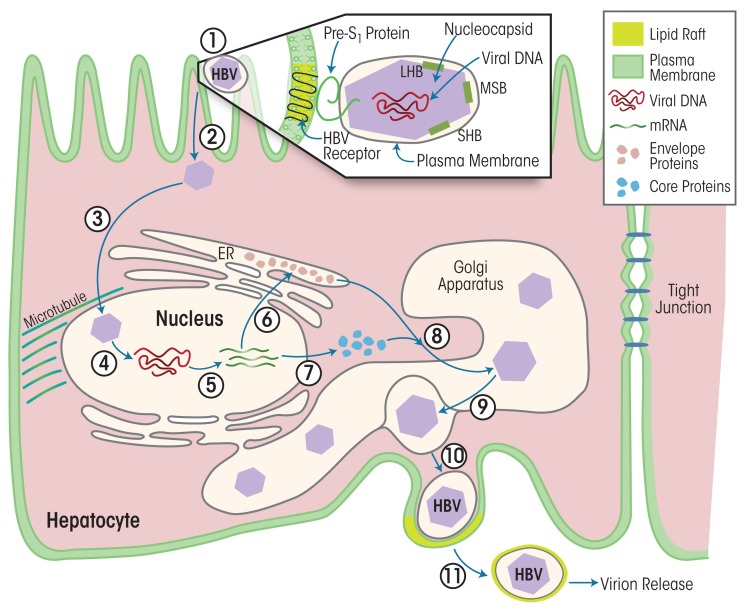
The life cycle of hepatitis B virus (HBV) and the role of lipid rafts. The HBV particles consist of an inner core particle (i.e., the nucleocapsid) that is made up of several envelope proteins, core proteins, and viral DNA. It is surrounded by a membrane derived from the previous host cell. **(1)** The virus particle attaches, presumably via the Pre-S1 protein, to unknown HBV receptors in the membrane of the cell. These receptors are located in membrane regions with characteristic lipid composition (i.e., lipid rafts). **(2)** The virus particle is taken up into the cell via a process called endocytosis and the nucleocapsid is released. **(3)** The nucleocapsid is transported into the nucleus, where **(4)** the DNA is released and **(5)** transcribed into mRNAs. **(6)** Some of the mRNAs are translated into the envelope proteins in the endoplasmic reticulum (ER). **(7)** Other mRNAs are translated into core proteins in the host cell’s cytosol. **(8)** Envelope proteins, core proteins, and mRNA move to the Golgi apparatus and assemble into immature core particles. **(9)** The immature particles mature in the Golgi apparatus, including reverse transcription of viral mRNA into DNA. **(10)** The mature particles become surrounded by the Golgi apparatus membrane. **(11)** The mature particles are released from the host cell, taking a piece of cellular membrane with them as an envelope, including lipid raft pieces.

HBV reproduces exclusively in hepatocytes. Each HBV particle contains a 3.2-kb open circular DNA encapsulated in a protein shell made of three envelope proteins and the enzymes HBV polymerase and cellular protein kinase C alpha (PKCα) (Wittkop et al. 2010). This complex is called the core particle or nucleocapsid. The nucleocapsid is further surrounded by a membrane derived from the previous host cell. When infecting cells, the viral envelope interacts with liver-specific receptors, leading to uptake into the cell (i.e., endocytosis) of the virus particle and release of the nucleocapsid (see figure 1). The nucleocapsid is transported to the nucleus, where the HBV genome is released and then transcribed into mRNAs that gives rise to three envelope proteins. In parallel, another viral mRNA is translated in the cytosol into the HBV core protein and viral polymerase. Then, the viral mRNA and the various viral proteins assemble to immature core particles in a membrane-enclosed cell structure called the Golgi apparatus. The HBV genomes mature within these core particles via reverse transcription of the pre-genomic mRNA to DNA. As soon as the mature virus is assembled, the viral particle release begins. Each virus particle is packaged into a cellular membrane coat from the Golgi apparatus and then released from the host cell, taking a bit of the cell membrane with it as an envelope.

Immune cells sense virus-infected cells, inducing a cytotoxic immune response. This response, combined with ongoing strong HBV DNA replication, often results in persistent, strong inflammatory disease (i.e., hepatitis), progressive fibrosis of the liver, and potentially in HCC (El-Serag 2012; Koziel 1998).

### HCV

**Figure 2:**
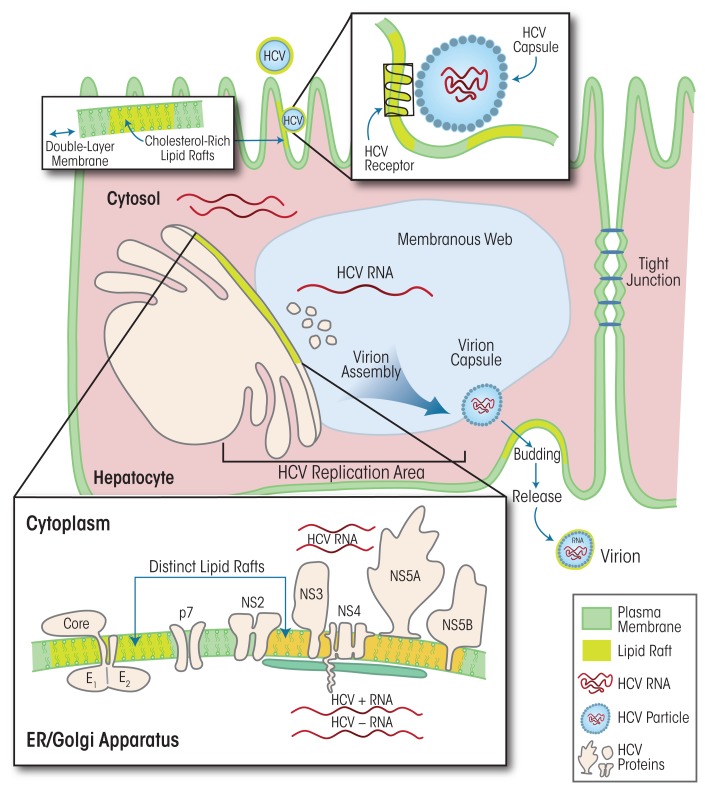
The life cycle of hepatitis C virus (HCV) and the role of lipid rafts. HCV particles attach to receptors in lipid-raft regions of the hepatocyte membrane, and the virus particles are taken up into the cell via endocytosis. The viral RNA is released and serves as template for the production of viral proteins at a structure called the membranous web, which also includes the membranes surrounding the endoplasmic reticulum (ER) and Golgi apparatus. The membranous web also is the site of assembly of new virus particles. During assembly and subsequent release of the viral particles, the particles obtain pieces of the cellular membrane as an outer envelope that shares the lipid composition of the membrane, particularly of the lipid rafts.

HCV is a positive-sense, single-stranded RNA virus that, like HBV, is thought to reproduce exclusively in hepatocytes (Paul and Bartenschlager 2014).^2^ HCV replicates in humans and high-level primates; it causes acute infections and has very high propensity to progress to chronic infection. The HCV viral particle includes the HCV RNA genome, the core, and an envelope made up of two glycoproteins (i.e., E1 and E2), which are key to the initial viral attachment to its cellular receptor/co-receptors (Flint and McKeating 2000; Rosa et al. 1996). Numerous molecules can serve as HCV receptors, such as scavenger receptor class B type I, low-density lipoprotein receptors, CD81, claudin-1, occludin, epidermal growth factor receptor, and Niemann-Pick C1-like 1 cholesterol absorption receptor (for a review, see Lindenbach and Rice 2013). Following attachment to the entry receptors, HCV is internalized into the host hepatocyte via endocytosis (Bartosch et al. 2003; Blanchard et al. 2006) and the RNA genome is released into the cytoplasm (see figure 2). The HCV RNA serves as template for the translation of a single large precursor protein that is processed further into 10 individual viral proteins. The translation, folding, processing, and function of these viral proteins depend on a specific intracellular structure in the hepatocytes called a membranous web, which also hosts viral RNA replication to generate new HCV genomes and assists in the assembly of new infectious viral particles (Chao et al. 2012). The assembly and release of these virus particles is closely linked to lipid metabolism (Paul et al. 2014). Thus, the lipid composition of the viral envelope is dependent on cholesterol biosynthetic pathways and resembles several types of cholesterol (i.e., low-density lipoprotein and very-low-density lipoprotein, with associated apolipoprotein E and/or B). In fact, the virus particles share the outer lipid coat with certain structures (i.e., lipid rafts, which will be discussed below) in the cell membrane surrounding the host hepatocytes (Chang et al. 2007; Gastaminza et al. 2008; Merz et al. 2011; Miyanari et al. 2007).

HCV replication is kept in check by the combined efforts of innate and adaptive (i.e., cellular and humoral) immune responses. In some people, the acute infections are mild and with limited clinical manifestations. In about 70 percent of infected individuals, however, the HCV infection is not cleared and a chronic infection is established. Possible mechanisms contributing to chronic HCV infection include failure of several types of immune cells, including natural killer cells, dendritic cells, and CD4 T cells (Dolganiuc et al. 2012; Koziel 2005; Szabo and Dolganiuc 2008). Persistently high viral replication that leads to steatotic transformation of hepatocytes and the subsequent death of some of the infected cells as well as an exaggerated inflammatory response to the infection can promote the development of fibrosis and induce disease progression from chronic hepatitis to end-stage liver disease and HCC.

## Synergistic Effects of HBV/HCV Infection and Alcohol Abuse on Liver Disease

### HBV and Alcohol Abuse

The prevalence of drinking in the general population is high, with more than 70 percent of people over age 18 in the United States reporting that they drank alcohol in the past year (National Institute on Alcohol Abuse and Alcoholism 2015). Accordingly, a significant portion of patients with chronic HBV infection are believed to have concomitant ALD. Alcohol use disorder is one of several conditions that may co-occur with chronic HBV infection and contribute to rapid progression of liver disease, increased likelihood of tumorigenesis, and accelerated progression of HCC (Ribes et al. 2006; Sagnelli et al. 2012). Thus, heavy alcohol intake in chronic HBV-infected patients is associated with a higher risk for developing liver cirrhosis; the prevalence of cirrhosis is about 2.5 times higher in patients with co-occurring HBV infection and alcohol abuse than in patients with only one of these conditions (Sagnelli et al. 2012). The prevalence of HCC and liver-related mortality also is higher in people with chronic HBV infection and concurrent heavy alcohol consumption (Hughes et al. 2011; Niro et al. 2010). Other co-occurring conditions that increase morbidity and mortality associated with chronic HBV infection and accelerate disease progression include infection with HCV, hepatitis D virus, and HIV (Ribes et al. 2006; Sagnelli et al. 2012).

Other studies found that alcohol promotes the presence of HBV particles in the blood (i.e., HBV viremia). For example, ethanol-fed mice showed up to sevenfold increases in the levels of HBV surface antigens (i.e., HBsAg) and viral DNA in the blood compared with mice fed a control diet. In addition, the ethanol-fed mice had elevated levels of HBV RNA as well as increased expression of various viral proteins (i.e., surface and core proteins) and X antigens in the liver (Larkin et al. 2001).

### HCV and Alcohol Abuse

The prevalence of chronic HCV infection is significantly elevated among people with alcohol use disorder (Fong et al 1994; Novo-Veleiro et al 2013) compared with the general population (prevalence of 1 to 2 percent) (CDC 2015*b*). Variables independently associated with HCV infection include female gender, injection drug use, and the presence of ALD (Novo-Voleiro et al. 2013). At the same time, patients with HCV infection have a higher prevalence of alcohol abuse and a longer duration of alcohol consumption compared with the general population (Degos 1999; Nevins et al. 1999; Pessione et al. 1998).

Chronic HCV infection results in the development of HCC in about 1 to 3 percent of patients after 30 years (Grebely and Dore 2011), contributing to the morbidity associated with HCV. The rate of HCC is substantially higher in people with HCV-related cirrhosis, reaching 2 to 4 percent per year in the United States, and even higher rates of up to 7 percent have been reported in Japan. Risk factors for the development of HCV-related HCC include male gender, age older than 55 years, and high levels of alcohol consumption (Grebely and Dore 2011; Hajarizadeh et al. 2013; Kim and Han 2012). Alcohol intake of 40 grams ethanol per day or more is associated with more rapid progression of liver disease associated with chronic HCV infection, including a more rapid increase in fibrosis and a doubled incidence of cirrhosis compared with patients with lower consumption levels (Wiley et al. 1998). Similarly, the risk of developing HCC is twice as high in patients with chronic HCV infection who drink heavily. Even small amounts of alcohol lead to an increased level of serum HCV RNA in patients with HCV infection (Cromie et al. 1996).

## Alcohol, Cellular Membranes, and Lipid Rafts

Biological membranes surround the cells and create compartments within the cells, such as the endoplasmic reticulum and Golgi apparatus. Current models view cellular membranes as tri-dimensional lipid–protein complexes that are easily disturbed. Thus, even small stimuli, such as changes in pH, ion environment, or binding of a molecule to a protein receptor, can lead to profound changes in the composition, function, and integrity of the cellular membrane. Not surprisingly, therefore, alcohol also can alter the state of the cellular membranes and may thereby affect cellular function. At the same time, proteins embedded in the cellular membranes may serve as receptors and points of entry for viruses, such as HBV and HCV.

The specific structure and function of hepatocyte membranes contributes to the ability of hepatitis viruses to infect the cells. In contrast to nonparenchymal liver cells, hepatocytes are polarized cells—that is, they have two clearly defined ends (i.e., an apical and a basolateral side), which is reflected in the membrane structure. Thus, the apical and basolateral membranes each have characteristic components that cannot mix, partially because the two cellular domains are separated by structures called tight junctions that also ensure the connection between a hepatocyte and its neighboring cells. The composition of polarized membranes differs between both ends of the cell with respect to both protein composition and lipid repertoire. Additionally, the membranes of both polarized and nonpolarized cells can be divided into lipid rafts and non–lipid-raft domains. Lipid rafts are membrane sections ranging in size from 10 to 200 nm that are enriched in specific lipids (i.e., sterols, sphingolipids, or ceramide). The specific structure of these lipid rafts promotes protein–protein and protein–lipid interactions; in addition, many cellular processes occur in these membrane regions. In both hepatocytes and other cell types, the overall protein concentration in the lipid rafts is relatively low, although certain proteins are highly concentrated in these membrane sections (Prinetti et al. 2000). The association with a lipid raft can influence the function of a protein (Paik et al. 2003; Pike 2006; Sonnino and Prinetti 2013). For example, proteins within lipid rafts are less able to move to other membrane areas, which favors more stable interactions with other proteins in the same domain. Thus, the activation of a cellular protein that serves as a receptor in a lipid raft facilitates clustering of the receptor with its co-receptors. Because the outer envelope of animal viruses such as HBV and HCV is derived from the host membranes, the lipid composition of the viral envelope resembles that of the membrane from which the virus buds (Laine et al. 1972). The cellular lipids and lipid rafts obtained from the host often modulate the membrane fusion between virus and host cell that is mediated by viral proteins (Teissier and Pécheur 2007) and therefore could become important targets for efforts to disrupt the viral life cycle. In general, the viruses seem to attach primarily to membrane areas containing lipid rafts; it remains to be determined whether viruses gain infectivity advantage if they attach to lipid rafts located in the apical or basolateral domain of the cell (Lindenbach and Rice 2013).

### Influence of Alcohol on Cellular Membranes and Lipid Rafts

The effect of alcohol exposure on cellular membranes, and lipid rafts in particular, depends on the cell type and its activity state as well as on the alcohol concentration and duration of exposure. It is important to remember, however, that alcohol’s effects on the cellular membrane do not occur in isolation; rather, they are part of alcohol’s global effects on the cell and on the tissue as a whole. In addition, liver-cell membranes may adapt to alcohol consumption (Rottenberg 1991), although it is difficult to determine which of those changes represent an adaptation and which represent pathological changes. Whether the adaptive changes of membrane composition, structure, and function delay or accelerate the onset of the pathological changes in the liver of human alcoholics also still is unclear.

**Figure 3:**
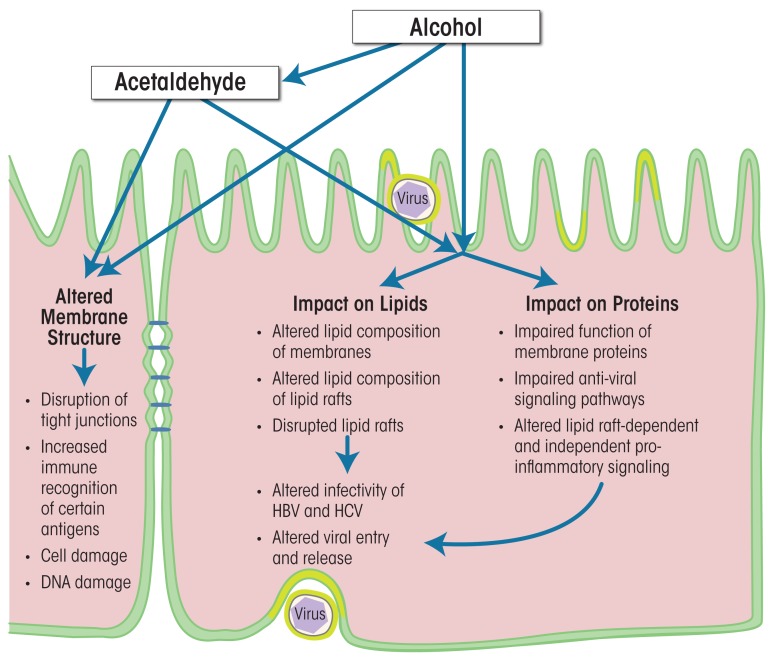
Alcohol’s effects on virus-infected hepatocytes. Alcohol may exert its effects both directly and indirectly. Indirect effects are, for example, related to the actions of the alcohol metabolite, acetaldehyde. Alcohol can directly affect both lipids and proteins in the cell. Through a variety of mechanisms, these effects may alter the infectivity of and the cell’s response to HBV and HCV, affecting both viral entry into the cells and release of viral particles from the cells.

Alcohol’s effects on cellular membranes can be indirect or direct (see figure 3). Indirect effects include, for example, the binding of acetaldehyde—which is a major metabolic product of ethanol and is found in high concentrations in the serum during alcohol abuse—to hepatocyte membranes. The acetaldehyde affects the structure of the cellular membrane, which leads to disruption of tight junctions, increased immune recognition of certain antigens, cell damage, DNA damage, and mutagenesis (Setshedi et al. 2010; Thiele et al. 2008). Alcohol’s direct effects on the cellular membrane can be subdivided further into effects on the lipids and effects on the protein components. Of these, alcohol’s effects on protein function probably have the greatest impact on both parenchymal and nonparenchymal liver cells. They occur during both the acute and the chronic phase of alcohol exposure and lead to significant functional impairment of the cells, which can cause cell death, tumorigenesis, altered intercellular communication, and increased susceptibility to additional insults, including viral infections. All of these can contribute to liver dysfunction. Studies have demonstrated that alcohol can impair the functions of proteins in cellular membranes and lipid rafts in liver cells in multiple ways, including actions on lipid-raft–associated signaling pathways (Dai and Pruett 2006; Dolganiuc et al. 2006). However, these studies have focused primarily on the outer cellular membrane and its lipid rafts; the effects of alcohol on intracellular lipid rafts (Chao 2012) remain to be characterized. Nevertheless, it is clear that as a result of the complex actions of alcohol on lipid-raft–associated signaling, the liver cells are more likely to create a proinflammatory milieu and downregulate their antiviral defense mechanisms. For example, studies have detected interference with signal transduction systems (Aliche-Djoudi et al. 2011; Dolganiuc et al. 2006; Nourissat et al. 2008) as well as enhancement of oxidative stress (Nourissat et al. 2008). Additionally, the cells spend excessive resources on efforts aimed at maintaining cellular homeostasis (e.g., remodeling the cellular membrane or re-ordering metabolic priorities) and on mechanisms to counteract cell death (Dolganiuc et al. 2012; Donohue and Thomes 2014). More importantly, exposure to alcohol, especially prolonged exposure, increases the liver cells’ vulnerability to second hits, including hepatitis viruses.

### Effects of Alcohol Abuse and Hepatitis Virus Infection on Cellular Membranes

As described above, the cellular membranes and lipid rafts are important targets of alcohol’s actions in the liver (Lieber 1980; Tsukamoto 1993) and are key in many aspects of alcohol-induced liver-cell dysfunction. Concurrent infection with HBV, HCV, and/or other viruses exacerbates alcohol’s detrimental effects on liver function and leads to an accelerated course of liver disease (Ribes et al. 2006; Tsui et al. 2006). The mechanism underlying the synergistic effects of hepatitis viruses and alcohol, and particularly the role of cellular membranes and lipid rafts, are not yet fully understood.

For HBV, alcohol’s effects on the membranes are relevant because the virus acquires its envelope from the membrane of the endoplasmic reticulum (Gerlich 2013). This envelope has a relatively high cholesterol content (Satoh et al. 2000), which is a key determinant of virus infectivity (Funk et al. 2007, 2008; Stoeckl et al. 2006). Thus, interference with the cellular membrane and lipid rafts during the viral life cycle, whether it is at the level of the host hepatocyte or cholesterol depletion from the virus membrane, has detrimental effects on the virus. Specifically, cholesterol-poor HBV virions take longer time to attach to, enter, and migrate inside the hepatocytes and are more likely to be cleared from the cells once they do enter (Funk et al. 2008). Alcohol exposure can distinctly alter the lipid composition of cellular membranes in general and lipid rafts in particular (Dolganiuc et al. 2006) and may thereby influence HBV infectivity. However, the precise effect of alcohol on the various steps of the HBV lifecycle remains largely unknown.

In addition to directly affecting both the virus and host parenchymal liver cells, alcohol influences anti-HBV immunity—an effect that also involves the cellular membrane as well as lipid rafts. HBV is known to interfere with normal T-cell function, and specifically with the T-cell receptor (TCR) that is responsible for recognizing and interacting with foreign antigens, thereby initiating an immune response. Thus, during HBV infection, the virus can impair the translocation of various components of the TCR (e.g., CD3f, ZAP-70, and Grb2) to lipid rafts; this is a hallmark of defective adaptive immune responses during chronic HBV infection (Barboza 2013; Larkin et al. 2001). Similarly, lipid-raft–dependent TCR localization and function are altered when adaptive immune cells are exposed to alcohol (Ishikawa et al. 2011). In particular, ethanol inhibits lipid-raft–mediated TCR signaling in CD4 T cells, resulting in suppression of IL-2 production (Ghare et al. 2011). Thus, alcohol acts synergistically with HBV to limit antiviral immunity. The consequences of alcohol’s effects on the TCR of HBV- and HCV-infected individuals are largely unknown but remain of high interest because adaptive immunity plays an important role in viral clearance (Barve et al. 2002; Heim and Thimme 2014; Loggi et al. 2014).

Compared with HBV, the life cycle of HCV depends on cellular membranes and lipid rafts to an even greater extent. HCV attaches to the cellular membrane and binds to a variety of cellular receptors that also serve as signaling molecules or receptors for other host proteins; most of these receptors reside in lipid rafts or are recruited there upon viral sensing and signaling. For example, one study found that compared with control cells, lipid rafts of cells expressing an HCV-1b genome showed altered levels of 39 proteins, including new or increased expression of 30 proteins and decreased expression of 9 proteins (Mannova and Beretta 2005). These alterations also affect a signaling pathway called the N-ras–PI3K–Akt–mTOR pathway (Peres et al. 2003; Zhuang et al. 2002); modulation of this pathway is one of the strategies by which HCV inhibits apoptosis and prevents elimination of infected cells. Alcohol can target these signaling platforms and may exert detrimental effects on lipid rafts that contain several putative HCV receptors, which may affect HCV replication and survival of HCV-infected cells. Thus, alcohol has been shown to affect the PI3K–mTOR pathway in non-liver cells (Li and Ren 2007; Umoh et al. 2014). However, the effect of alcohol on the PI3K–mTOR pathway in parenchymal and nonparenchymal liver cells remains to be determined.

Alcohol also adversely affects many of the immune cells and pathways that are considered key to antiviral immunity to HCV. Thus, alcohol exposure enhances signaling via TLRs that mediate inflammation and impairs signaling via TLRs that mediate production of antiviral molecules, including interferons. Of note, some of the same pathways are targeted in similar ways by HCV, thus producing synergistic effects that promote inflammatory reactions and support the viral lifecycle in both parenchymal and non-parenchymal liver cells (John and Gaudieri 2014; Koziel 2005; Pang et al. 2011; Szabo et al. 2010).

## Conclusions

Alcohol exposure and hepatitis viruses exploit common mechanisms to promote liver disease. Some of these mechanisms focus on the cellular membrane and its most active domains, the lipid rafts, which play critical roles in sustaining the lifecycle of both HBV and HCV. For HBV, the cellular membranes and lipid rafts are particularly involved in viral entry; for HCV, lipid rafts additionally are required for formation and/or maintenance of HCV viral replication, virion assembly, and virion release from the host cell. Lipid rafts additionally influence viral survival indirectly because they serve as signaling platforms for a proinflammatory signaling cascade as well as for antiviral pathways, and they help regulate intracellular lipid storage within the parenchymal liver cells. Moreover, cellular membranes and lipid rafts play a crucial role in the immune-mediated cell defense in nonparenchymal liver cells. Alcohol affects membrane and lipid-raft function both directly and indirectly by modulating the proinflammatory cascade as well as antiviral pathways and intracellular lipid storage within the parenchymal liver cells and hampering the function of nonparenchymal liver cells through both lipid-raft–dependent and –independent mechanisms. The synergistic effects of hepatitis viruses and alcohol on the cellular membranes lead to impaired antiviral immunity and a proinflammatory milieu in the liver, thereby helping to sustain the viral lifecycle and promoting rapid progression and a more severe course of liver disease.

A better understanding of lipid-raft function may contribute to new approaches to treatment of viral and alcohol-related hepatitis, but knowledge of the structure and function of these cell structures is only beginning to emerge. For example, lipid-raft formation still is an enigma, and researchers are only now starting to investigate and understand the processes underlying lipid-raft activation, protein–lipid interactions, lipid-raft–dependent signaling, and other mechanisms through which lipid rafts direct the bioactivity of the various membrane constituents. Eventually, however, better understanding of cellular membranes and lipid rafts and their involvement in health and disease may lead to novel treatment approaches, including cellular- and subcellular-based personalized medicine approaches that also may lead to improved outcomes for patients with viral and/or alcohol-related hepatitis.

## Footnotes

1 The hepatitis A virus usually only causes self-limiting infections. Hepatitis D virus requires the helper function of HBV to replicate and thus hepatitis D virus infections only occur in people infected with HBV. Hepatitis E virus primarily is found in Asia and Africa and is less common in Europe and the Americas. Chronic hepatitis E virus infection only has been observed in people receiving immunosuppressant treatment after an organ transplant. The effects of alcohol on hepatitis A, D, and E viruses are largely unknown.

2 Some reports suggest that viral replication outside the liver may also occur (Revie and Salahuddin 2011).
